# Exosomal lncRNA TUG1 from cancer-associated fibroblasts promotes liver cancer cell migration, invasion, and glycolysis by regulating the miR-524-5p/SIX1 axis

**DOI:** 10.1186/s11658-022-00309-9

**Published:** 2022-02-22

**Authors:** Le Lu, Jingjing Huang, Jiantao Mo, Xuanbo Da, Qiaoxin Li, Meng Fan, Hongwei Lu

**Affiliations:** Department of General Surgery, The Second Affiliated Hospital of Xi’an Jiaotong University, No.157, West 5th Road, Xi’an, 710004 China

**Keywords:** Long noncoding RNA, Taurine upregulated gene 1, Hepatocellular carcinoma, microRNA, Sine oculis homeobox homolog 1, Exosomes

## Abstract

**Background:**

Increasing evidence suggests that taurine upregulated gene 1 (TUG1) is crucial for tumor progression; however, its role in hepatocellular carcinoma (HCC) and the underlying mechanisms are not well characterized.

**Methods:**

The expression levels of TUG1, miR-524-5p, and sine oculis homeobox homolog 1 (SIX1) were determined using quantitative real-time PCR. The regulatory relationships were confirmed by dual-luciferase reporter assay. Cell proliferation and invasion were assessed using Cell Counting Kit 8 and transwell assays. Glucose uptake, cellular levels of lactate, lactate dehydrogenase (LDH), and adenosine triphosphate (ATP) were detected using commercially available kits. Silencing of TUG1 or SIX1 was performed by lentivirus transduction. Protein levels were measured by immunoblotting.

**Results:**

Cancer-associated fibroblasts (CAFs)-secreted exosomes promoted migration, invasion, and glycolysis in HepG2 cells by releasing TUG1. The promotive effects of CAFs-secreted exosomes were attenuated by silencing of TUG1. TUG1 and SIX1 are targets of miR-524-5p. SIX1 knockdown inhibited the promotive effects of miR-524-5p inhibitor. Silencing of TUG1 suppressed tumor growth and lung metastasis and therefore increased survival of xenograft model mice. We also found that TUG1 and SIX1 were increased in HCC patients with metastasis while miR-524-5p was decreased in HCC patients with metastasis.

**Conclusions:**

CAFs-derived exosomal TUG1 promoted migration, invasion, and glycolysis in HCC cells via the miR-524-5p/SIX1 axis. These findings may help establish the foundation for the development of therapeutics strategies and clinical management for HCC in future.

**Supplementary Information:**

The online version contains supplementary material available at 10.1186/s11658-022-00309-9.

## Background

Hepatocellular carcinoma (HCC) is the fourth most common cause of cancer-related deaths worldwide [1]. The incidence rates of HCC are particularly high in Asia and Africa [2]. According to the World Health Organization, an estimated 1 million people are projected to die from liver cancer in 2030 [3]. Chronic viral hepatitis, alcohol consumption, diabetes, and nonalcoholic steatohepatitis are the main risk factors for the development of HCC [4, 5]. Treatment of HCC is typically challenging and recurrence is a major problem after curative treatment [6]. Therefore, development of improved therapeutics for HCC is imperative. Exosomes can be produced by different types of cells [7]. The exosomal content is heterogeneous and generally includes proteins, lipids, DNAs, mRNAs, lncRNA, and miRNA [8, 9]. Recent studies have shown that exosomes contribute to cancer progression by transferring different types of substances [10, 11].

lncRNAs are defined as noncoding RNAs longer than 200 nucleotides. lncRNAs regulate gene expression and have been implicated in cancer. A previous study showed that lncRNAs may promote proliferation, migration, invasion, and metastasis of liver cancer [6, 12]. miRNAs are small noncoding RNAs (∼ 22 nt) that play an important role in regulating gene expression. miR-524-5p has been implicated in various diseases. Liu et al. reported that miR-524-5p suppresses skin cancer [13]. Li et al. found that miR-524 suppresses angiogenesis in colorectal cancer [14].

LncRNAs can act as miRNA sponges, thereby reducing the regulatory effect of miRNAs on their target mRNAs. Several studies have implicated both lncRNAs and miRNAs in the development of cardiovascular diseases and cancer [15–17]. LncRNA taurine upregulated gene 1 (TUG1) has been implicated in oncogenesis [18–20]. TUG1 was found to promote the progression of colorectal cancer and laryngeal cancer [21, 22]. In a recent study, lncRNA TUG1 was shown to control the progression of oral cancer via miR-524/DLX1 [23].

Sine oculis homeobox homolog 1 (SIX1) is a homeodomain-containing transcription factor. SIX1 is highly expressed during embryo development, plays important roles in mammalian organ development, enhances progenitor cell survival, and its absence leads to the reduction in size or loss of many organs [24]. SIX1 is upregulated in human tumor tissues, and its expression levels are negatively correlated with immune cell infiltration in the tumor microenvironment and the overall survival rates of cancer patients [25]. SIX1 has been identified as a therapeutic target for HCC owing to its involvement in tumor onset and progression [26, 27]. SIX1 directly regulates expression of glycolytic genes such as HK2 and PKM2, glucose uptake, and the level of lactate, a metabolite that can modulate tumor cell proliferation, apoptosis, and metastasis [28]. In this study, we investigated the role of cancer-associated fibroblasts (CAFs)-derived exosomal TUG1, SIX1 and miR-524-5p in the migration, invasion, and glycolysis in HCC cells.

## Materials and methods

### Ethics statement and clinical samples

Fresh tumor tissues and adjacent non-tumor tissues with normal liver architecture after histological examination by two independent pathologists were collected from 120 HCC patients who underwent surgery between January 2018 and December 2019 at the Second Affiliated Hospital of Xi’an Jiaotong University. The clinicopathological characteristics and follow-up data of patients with HCC are shown in Additional file 1: Table S1. None of these patients had received radiotherapy or chemotherapy prior to surgery. We performed the study following the principles of the Declaration of Helsinki. The study was approved by the Ethics Committee of The Second Affiliated Hospital of Xi’an Jiaotong University Ethics Committee (approval no. 2017060, date: 2017.7.18). Written informed consent was obtained from all patients.

### Cell culture

Fibroblasts from liver cancer (CAFs) or adjacent non-tumor liver tissues (NFs) were isolated as mentioned previously [29]. CAFs were cultured in DMEM/F12 with 10% FBS (Invitrogen, Shanghai, China) at 37 °C. Primary fibroblasts used in this study were between passages 3 and 5. HepG2 cells were obtained from the Shanghai Biology Institute (Shanghai) and cultured in DMEM (Invitrogen, Shanghai) with 10% FBS and antibiotics at 37 °C.

### Isolation and experimental analysis of exosomes

Cells were washed with PBS and cultured in complete medium with exosome-free FBS for 2 days. Medium was centrifuged at 2200 *g* for 15 min and 11,000 *g* for 35 min, followed by filtration with a 0.22-mm filter. Medium was then centrifuged at 110,000 *g* for 100 min. Pellets were re-suspended and centrifuged at 110,000 *g* for 100 min and re-suspended in 50 μL of PBS. Exosomes were placed on a copper grid for examination under an electron microscope.

Exosomes were labeled with PKH67 (Sigma, Shanghai) and co-cultured with HepG2 for 24 h. The uptake of exosomes by HepG2 was analyzed with an Olympus FV1200 microscope.

### Co-culture assay

HepG2 cells (5 × 10^4^) were seeded in lower chambers, and the same amount of fibroblasts was seeded on the Transwell membranes. Fibroblasts were treated with or without GW4869 for 24 h before seeding. After 6 days of co-culture, HepG2 cells were used for RNA and protein extraction or further cytological experiments.

### miRNA transfection

miR-524-5p mimic (5′-CUACAAAGGGAAGCACUUUCUC-3′), miR-524-5p inhibitor (5′-GAGAAAGUGCUUCCCUUUGUAG-3′), and miR-NC (5′-CAGUACUUUUGUGUAGUACAA-3′) were synthesized by Beyotime (Beijing, China) and transfected into cells using Lipofectamine 2000 (Invitrogen, Shanghai).

### Plasmid, lentivirus and adenovirus construction

The TUG1 encoding sequence was cloned into a pcDNA3.1(+) vector (Addgene, USA) to obtain pcDNA3.1(+)-TUG1 expressing vector. RNA interference sequences targeting TUG1 or SIX1 were introduced into pLKO.1 plasmids (Addgene), respectively. The sequences were: shTUG1-1: 5′-CCTTGTTTAGTGCATCTTT-3′; shTUG1-2: 5′-CCCACATACACCACAACAT-3′; shSIX1: 5′-GCAACTTCCGTGAGCTCTA-3′. The 293T cells were plated in 6-well plates and transfected with the constructs for 4–6 h using Lipofectamine 2000 reagent (Invitrogen) as well. Scramble shRNA (shNC) and empty vector, respectively, were used as the negative control.

### In vitro exosome supplementation

HepG2 cells were provided with fresh media plus 100 μg/mL exosomes isolated from CAFs for 48 h.

### Cell proliferation analysis

Cell proliferation was assessed using a CCK-8 kit (Biovision, Exton, PA). HepG2 cells were cultured in a 96-well plate. CCK-8 (10 μL) was added to each well for 2 h. Subsequently, the optical absorbance at 450 nm was recorded with a microplate reader (Promega, Madison, WI).

### Transwell assay

After the aforementioned treatment, HepG2 cells in a serum-free medium were cultured in the upper transwell chamber (Corning, Tewksbury, MA) equipped with an 8.0 μm pore polycarbonate membrane. DMEM with 10% FBS was added to the lower chamber. After 48 h of incubation, cells that had migrated through the membrane and adhered to the lower surface of the membrane were fixed and stained with crystal violet. Studies of invasion were performed as described previously with the exception that the membranes utilized were Matrigel-coated invasion chambers (BD Biosciences, San Jose, California, USA).

### Measurement of glucose uptake

Glucose uptake was measured using a glucose uptake assay kit (Biovision, Exton, PA). After the aforementioned treatment, HepG2 cells were starved for glucose for 3 h. After incubation in Krebs–Ringer Bicarbonate Buffer with 2% bovine serum albumin for 40 min, 2-NBDG (100 µM) was injected into each well and the cells were incubated for 50 min at 37 °C. Cells were washed, trypsinized, re-suspended in 10% FBS, and analyzed by flow cytometry (Thermo Fisher, Waltham, MA).

### Measurement of lactate, lactate dehydrogenase (LDH), and adenosine triphosphate (ATP)

After the aforementioned treatment, cellular lactate release, ATP level, and cellular LDH activity of HepG2 cells were measured using commercially available kits (Jiancheng Bio. Nanjing).

### Luciferase reporter assay

TUG1 sequences harboring a putative miR-524-5p binding site and 3′-UTR sequences of SIX1 as well as their mutant sequences were inserted into the pGL3 vector (Promega). Then, the wild-type (WT) or mutant (Mut) pGL3-TUG1 or pGL3-SIX1 and pRL-TK *Renilla* (Promega) luciferase reporter vector were cotransfected into the HepG2 cells, which were transfected with miR-524-5p mimic or miR-NC. Luciferase activity was assessed using a Dual-Luciferase Reporter Assay system (Promega) at 2 days after transfection.

### RNA isolation and quantitative RT-PCR (qRT-PCR) analyses

Total RNA was extracted using TRIzol and reverse transcribed using Superscript II (Invitrogen, Shanghai). SYBR master mix (Bio-Rad, Philadelphia, PA) was used for qRT-PCR. Stem-loop real-time RT-PCR was carried out to analyze miRNA expression. Briefly, extracted RNAs were converted into cDNAs with a cDNA synthesis kit (Thermo Fisher Scientific). qRT-PCR was then performed using Maxima SYBR Green qPCR Master Mixes (Thermo Fisher Scientific) according to the manufacturer’s instructions. *GAPDH* or *U6* was used as the internal reference gene. The primers are listed in Additional file 1: Table S2.

### Western blotting

Proteins were extracted and quantified using a BCA Kit (Beyotime, Beijing). Proteins were resolved by 10% SDS-PAGE, transferred to PVDF membranes (Bio-Rad, Philadelphia, PA), blocked, and incubated with primary antibodies against MMP-2 (ab97779; Abcam, Cambridge, MA, USA), MMP-9 (#2270; Cell Signaling Technology, Danvers, MA, USA), HK2 (ab104836), LDHA (ab125683), SIX1 (ab252224), TSG101 (ab125011), CD9 (ab92726), CD63 (ab216130), or GAPDH (ab9485). After washing three times, the membranes were incubated with secondary antibodies (A0208, A0216; Beyotime, Shanghai, China), visualized by an ECL kit (Bio-Rad, Philadelphia, PA), and the results analyzed by Image-Pro6.0 software.

### RNA immunoprecipitation (RIP) assays

RNAs were immunoprecipitated (IP) using a Magna RIP RNA-Binding Protein Immunoprecipitation kit (Millipore) following the manufacturer’s instructions. Briefly, cells were lysed in RIP lysis buffer, and RNA magnetic beads were conjugated with anti-AGO2 (Abcam, ab186733) or anti-IgG antibody (Abcam, ab172730) overnight at 4 °C and washed with RIP-wash buffer for 10 min at 4 °C and then RIP-lysis buffer for 5 min at 4 °C. The coprecipitated RNAs were used for cDNA synthesis and evaluated by qRT-PCR as described above.

### In vivo model

Male BALB/c nude mice were obtained from Zhengzhou University. This study was approved by the Ethics Committee of The Second Affiliated Hospital of Xi'an Jiaotong University (approval no. 2017061, date: 2017.7.18). BABL/c nude mice were housed in individually ventilated cages under specific pathogen‑free conditions including a 24‑h light/dark cycle, 25 °C temperature, and 80% humidity. Mice were provided access to sterilized water and food ad libitum. After the aforementioned treatment, HepG2 cells (5 × 10^6^) were injected into each mouse through the tail vein. Exosomes (10 μg) derived from CAFs with control pShuttle-H1 adenovirus (CAFs/shNC-exo) or TUG1 shRNA adenovirus (Obio Technology Company, Shanghai, China) (CAFs/shTUG1-exo) were then injected into tail veins every 3 days. Therefore, mice were divided into three groups: the blank group (HepG2 cell injection), CAFs/shNC-exo group (HepG2 cell and CAFs/shNC-exo injection) and CAFs/shTUG1-exo group (HepG2 cell and CAFs/shTUG1-exo injection). Five weeks after injection, 18 mice were anesthetized by inhalation with 3% isoflurane, sacrificed by cervical dislocation, and liver tissues were harvested for counting of tumor nodules and hematoxylin and eosin (H&E) staining (n = 6 per group). 45 other mice were used for survival analysis for 84 days (n = 15 per group).

### Data analysis

Experiments were performed in triplicate. Data analysis was performed using GraphPad Prism 8.4.2 (San Diego, CA). Results are presented as mean ± standard deviation (SD). Between-group differences were assessed using Student’s *t* test or one-way ANOVA followed by Tukey’s post-multiple test. The Kaplan–Meier method and Cox’s proportional hazards regression model were used to calculate overall survival, and differences between the groups were analyzed by the log-rank test. P values < 0.05 were considered indicative of statistical significance.

## Results

### CAFs promoted migration, invasion, and glycolysis in HepG2 cells

**Fig. 1:**
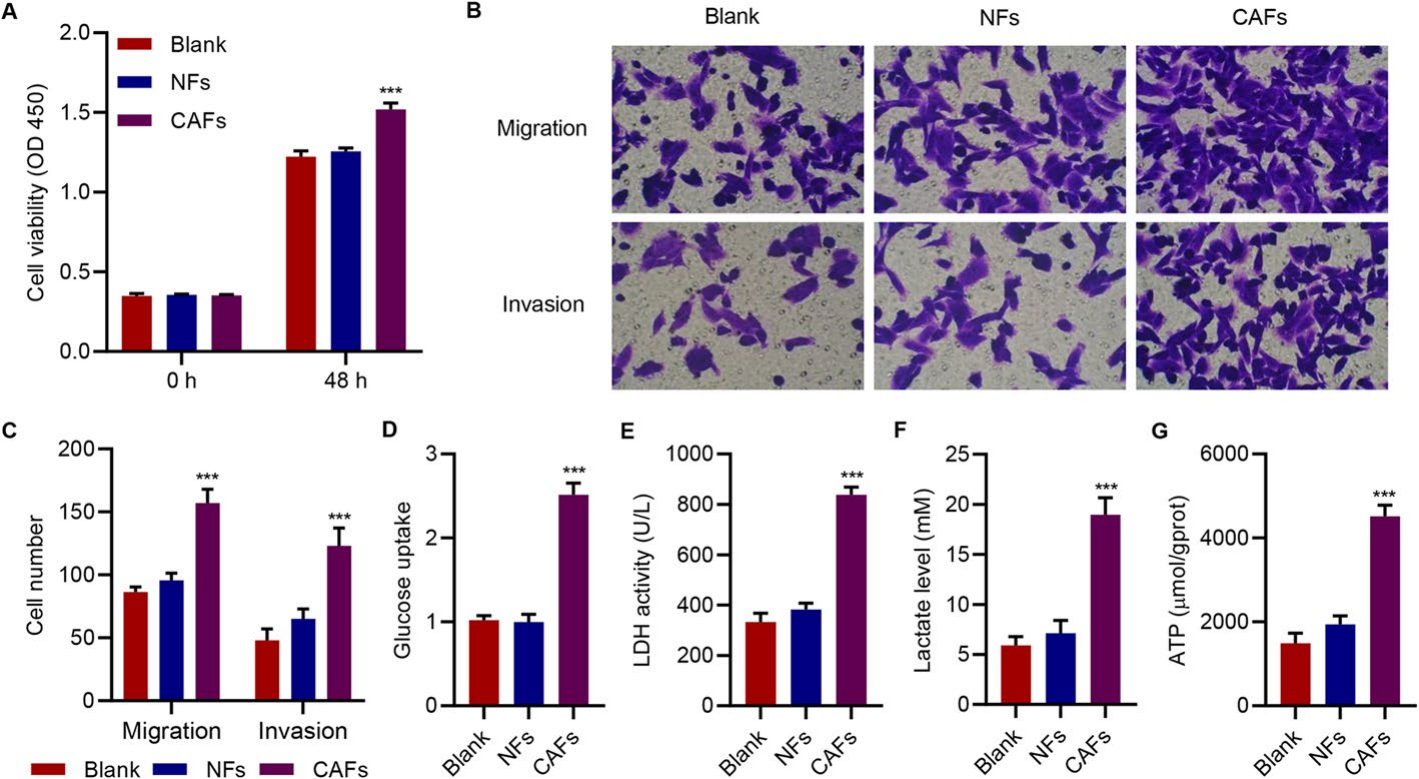
CAFs promoted migration, invasion, and glycolysis in HepG2 cells. Effects of CAFs and NFs on the (**A**) viability, (**B**, **C**) migration, and (**B**, **C**) invasion of HepG2 cells. Effects of CAFs and NFs on (**D**) glucose uptake, (**E**) LDH activity, (**F**) lactate levels, and (**G**) ATP content of HepG2 cells. The data are expressed as the mean ± SD (n = 3). ***P < 0.001 compared with blank (HepG2 cells without treatment)

In order to study the role of CAFs in cell migration, invasion and glycolysis, CAFs and NFs were isolated from tumor tissues and adjacent non-tumor tissues and co-cultured with HepG2 cells. Both CAFs and NFs demonstrated positive staining for the specific fibroblast marker vimentin and negative staining for the epithelial marker CK19, and CAFs expressed vimentin more significantly (Additional file 1: Figure S1). The results showed that, compared with controls, administration of CAFs resulted in a significant increase in cell viability (Fig. 1A), migration, and invasion (Fig. 1B, C). Glycolysis is related to multiple reactions such as glucose uptake, converting pyruvate to lactate by lactate dehydrogenase (LDH), and adenosine triphosphate (ATP) production [30]. Thus, we investigated whether CAFs affected the glycolysis of HepG2 cells. Administration of CAFs also caused a significant increase in glucose uptake (Fig. 1D), LDH activity (Fig. 1E), lactate levels (Fig. 1F), and ATP levels in HepG2 cells (Fig. 1G). However, NFs did not demonstrate these effects. These findings indicated that CAFs promoted migration, invasion, and glycolysis in HepG2 cells.

### CAFs-derived exosomes promoted migration, invasion, and glycolysis in HepG2 cells

**Fig. 2:**
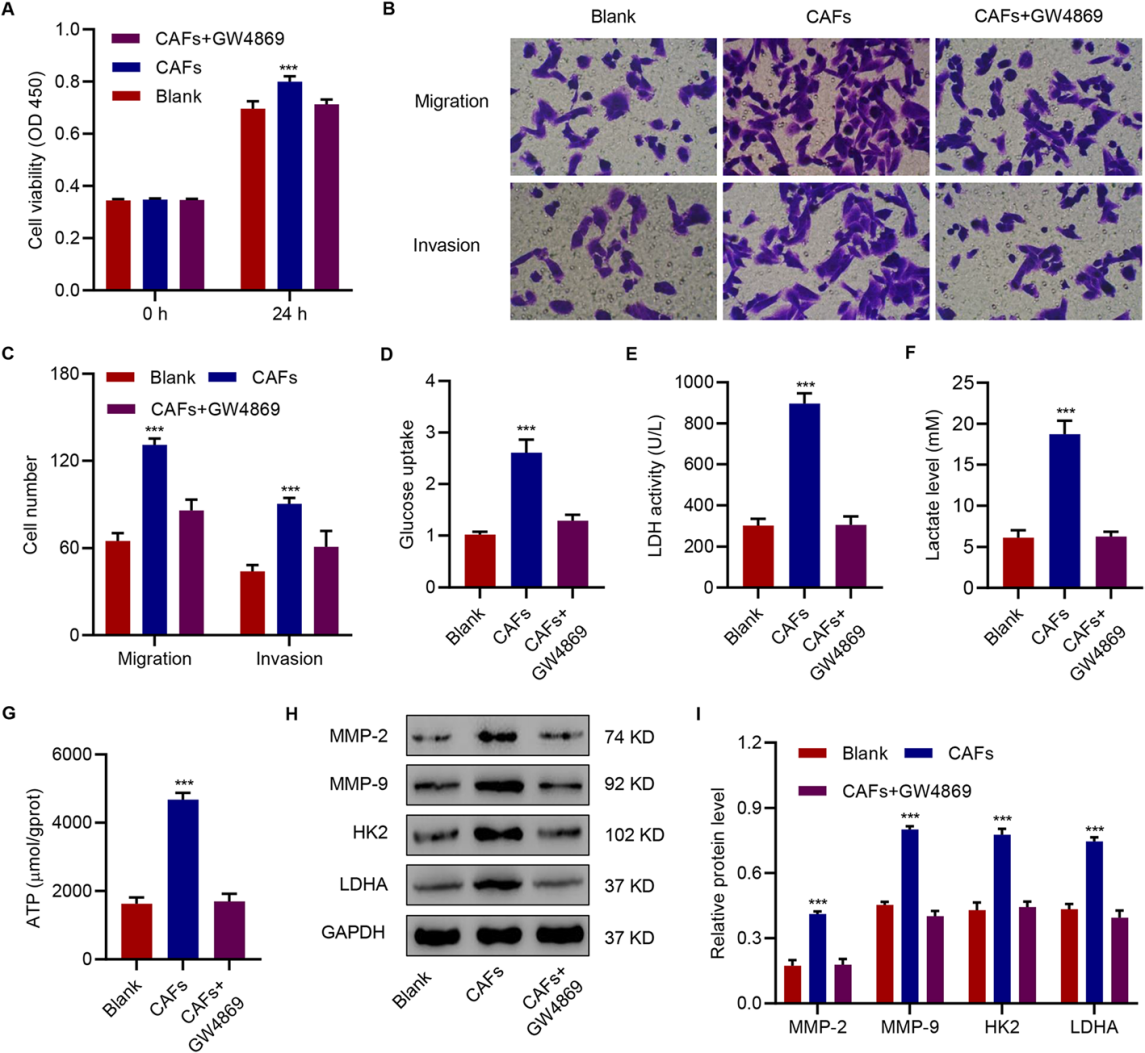
CAFs-derived exosomes promoted migration, invasion, and glycolysis in HepG2 cells. Effects of CAFs with or without GW4869 on the (**A**) viability, (**B**, **C**) migration and invasion, (**D**) glucose uptake, (**E**) LDH activity, (**F**) lactate, (**G**) ATP content, and (**H**, **I**) MMP-2, MMP-9, HK2, and LDHA of HepG2 cells. The data are expressed as the mean ± SD (n = 3). ***P < 0.001 compared with blank (HepG2 cells without treatment)

In order to study the mechanism by which CAFs promoted migration, invasion, and glycolysis in HCC cells, CAFs with or without exosome inhibitor GW4869 treatment were co-cultured with HepG2 cells. The results showed that CAFs resulted in a significant increase in cell viability (Fig. 2A), migration, and invasion (Fig. 2B, C), glucose uptake (Fig. 2D), LDH activity (Fig. 2E), lactate levels (Fig. 2F), ATP levels (Fig. 2G), and levels of MMP-2, MMP-9, HK2, and LDHA in HepG2 (Fig. 2H, I). However, the treatment of HepG2 cells with CAFs and GW4869 did not show significant effects on the measured parameters. These data suggested that CAFs promoted migration, invasion, and glycolysis in HepG2 cells through exosomes. Next, exosomes isolated from culture medium of CAFs were used to treat HepG2 cells. CAFs-derived exosomes (CAFs-exo) were first examined by transmission electron microscopy (TEM) (Additional file 1: Figure S2A); subsequently, these were subjected to Western blotting for detection of exosome markers CD63, CD9, and TSG101 (Additional file 1: Figure S2B). Confocal microscopy confirmed the internalization of exosomes by the HepG2 cells (Additional file 1: Figure S2C).

### TUG1 transferred from CAFs by exosomes promoted migration, invasion, and glycolysis in HepG2 cells

**Fig. 3:**
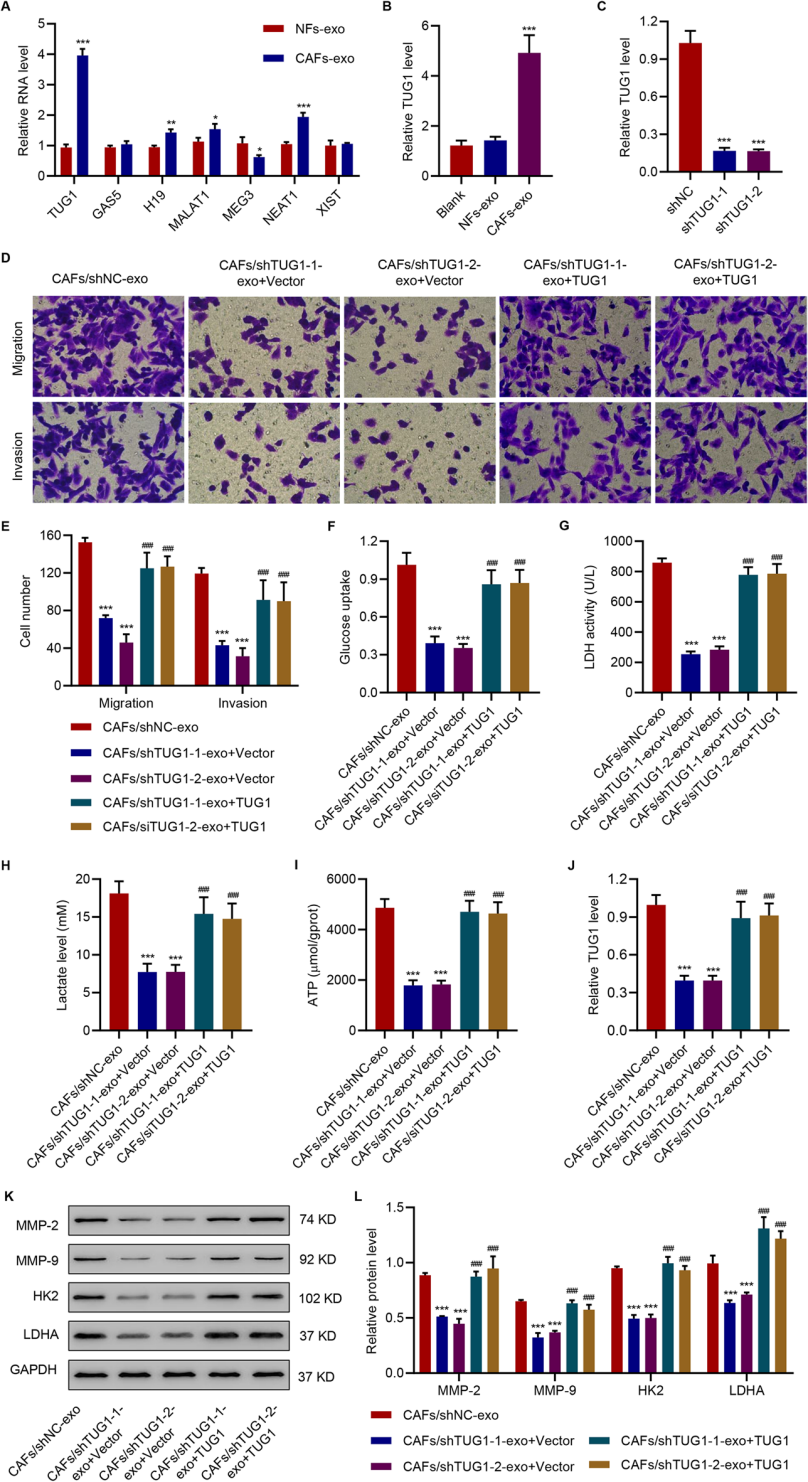
CAFs-derived exosomal TUG1 promotes migration, invasion, and glycolysis in HepG2 cells. **A** Expression levels of TUG1, GAS5, H19, MALAT1, MEG3, NEAT1, and XIST in NFs-exo and CAFs-exo as measured by qPCR. **B** TUG1 level in HepG2 treated with NFs-exo or CAFs-exo determined by qPCR. **C** TUG1 level in exosomes derived from the CAFs transduced with TUG1 shRNA or shNC determined by qPCR assay. Effects of exosomes derived from CAFs transduced with TUG1 shRNA or shNC, and TUG1 overexpression in HepG2 cells on (**D**, **E**) migration and invasion, (**F**) glucose uptake, (**G**) LDH activity, (**H**) lactate, and (**I**) ATP content, (**J**) TUG1 expression, and (**K**, **L**) MMP-2, MMP-9, HK2, and LDHA. The data are expressed as the mean ± SD (n = 3). **P < 0.01, ***P < 0.001 vs NFs-exo, blank (HepG2 cells without treatment), shNC or CAFs/shNC-exo. ^###^*P* < 0.001 vs corresponding CAFs/shTUG1-exo + Vector

To determine the mechanism by which CAFs-exo promoted migration, invasion, and glycolysis in HepG2 cells, we analyzed the expressions of TUG1, H19, MALAT1, NEAT1, and MEG3 using RT-PCR. We found that compared with NFs-exo, CAFs-exo exhibited higher expression levels of *TUG1* (P < 0.0001), *H19* (P = 0.0021), *MALAT1* (P = 0.0285), and *NEAT1* (P = 0.0006), and a lower expression level of *MEG3* (P = 0.0224) (Fig. 3A). Compared with other lncRNAs with increased expression, TUG1 expression was highest in CAFs-exo, increased in HCC patients, and associated with survival outcome [31]. TUG1 was therefore selected for the following experiments. To further investigate the role of CAFs-exo and TUG1 in the metastatic potential of HCC cells, HepG2 cells were treated with exosomes from CAFs transduced with TUG1 shRNA (shTUG1) or negative control (shNC). CAFs-exo remarkably enhanced TUG1 in HepG2 cells (Fig. 3B) and TUG1 was successfully silenced by shRNA and measured in CAFs-exo (Fig. 3C). The exosomes from CAFs/shTUG1 or CAFs/shNC were used to treat HepG2 cells. The results showed that silencing of TUG1 in CAFs significantly ameliorated the effects of CAFs-exo on migration and invasion (Fig. 3D, E). To study the function of TUG1 in HepG2 development in vivo, we inoculated mice with HepG2 and administered exosomes derived from CAFs with or without TUG1 shRNA adenovirus infection to tumor-bearing mice. H&E staining showed that silencing of TUG1 suppressed the promotive effect of CAFs-exo on lung metastasis (Additional file 1: Figure S3A–3B); this led to enhanced survival of mice injected with CAFs-exo (Additional file 1: Figure S3C). Moreover, silencing of TUG1 in CAFs also significantly ameliorated the effects of CAFs-exo on glucose uptake (Fig. 3F), LDH activity (Fig. 3G), levels of lactate and ATP (Fig. 3H, I), and expression levels of TUG1, MMP-2, MMP-9, HK2, and LDHA in HepG2 cells (Fig. 3J–L). However, overexpression of TUG1 in HepG2 cells significantly ameliorated the effect of exosomes derived from shTUG1 transduced CAFs on HepG2 cells (Fig. 3D–L).

Next, CAFs-exo were used to treat HepG2 cells transduced with either shTUG1 or shNC. The results showed that silencing of TUG1 significantly suppressed the effects of CAFs-exo on migration, invasion, and glycolysis in HepG2 cells (Additional file 1: Figure S4A–I). Taken together, these results indicated that the effects of CAFs-exo were mediated by TUG1.

### TUG1 attenuated miR-524-5p mimic-mediated anti-tumor effects in HepG2 cells

**Fig. 4:**
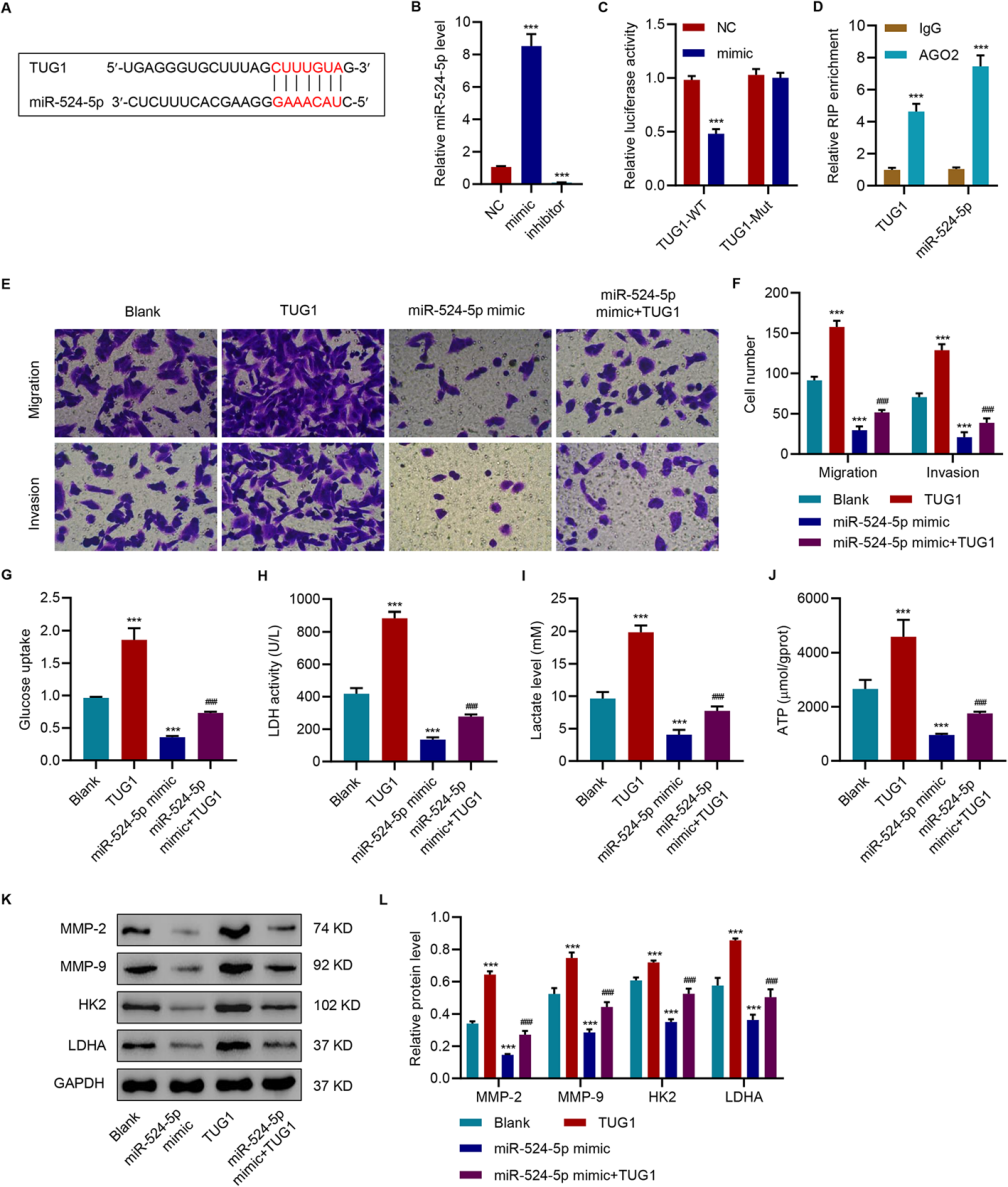
TUG1 attenuates miR-524-5p mimic-mediated anti-tumor effects in HepG2. **A** The predictive binding of miR-524 and TUG1. Relative levels of miR-524 **B** in HepG2 cells with overexpression or silencing of miR-524-5p. **C** Binding of miR-524 and TUG1. **D** RIP experiments were performed in HepG2 cells with an AGO2 antibody, and the co-precipitated RNA was subjected to qPCR for TUG1. Effects of TUG1 overexpression and miR-524 mimic on (**E**, **F**) migration and invasion, (**G**) glucose uptake, (**H**) LDH activity, (**I**) lactate, and (**J**) ATP content, and (**L**, **M**) expression of MMP-2, MMP-9, HK2, and LDHA. The data are expressed as the mean ± SD (n = 3). ***P < 0.001 vs. blank or NC. ^###^*P* < 0.001 compared with miR-524 mimic

We performed bioinformatics analysis to investigate the mechanism by which TUG1 mediated the effects of CAFs-exo; the results showed potential binding between TUG1 and miR-524-5p (Fig. 4A). Bioluminescent experiments indicated that overexpression of miR-524 remarkably inhibited the luciferase activity of WT TUG1 (Fig. 4B, C). The results in Fig. 4D show that TUG1 and miR-524-5p were highly enriched with the AGO2 antibody in HepG2 cells. Moreover, TUG1 overexpression ameliorated the effects of miR-524-5p mimic on migration and invasion (Figs. 4E, F), glucose uptake (Fig. 4G), LDH activity (Fig. 4H), lactate levels (Fig. 4I), ATP levels (Fig. 4J), and expression levels of MMP-2, MMP-9, HK2, and LDHA in HepG2 (Fig. 4K, L). These results suggested that TUG1 functions as a miR-524-5p sponge to regulate migration, invasion, and glycolysis in HepG2 cells.

### SIX1 knockdown attenuated miR-524-5p inhibitor-mediated promotion of migration, invasion, and glycolysis in HepG2 cells

**Fig. 5:**
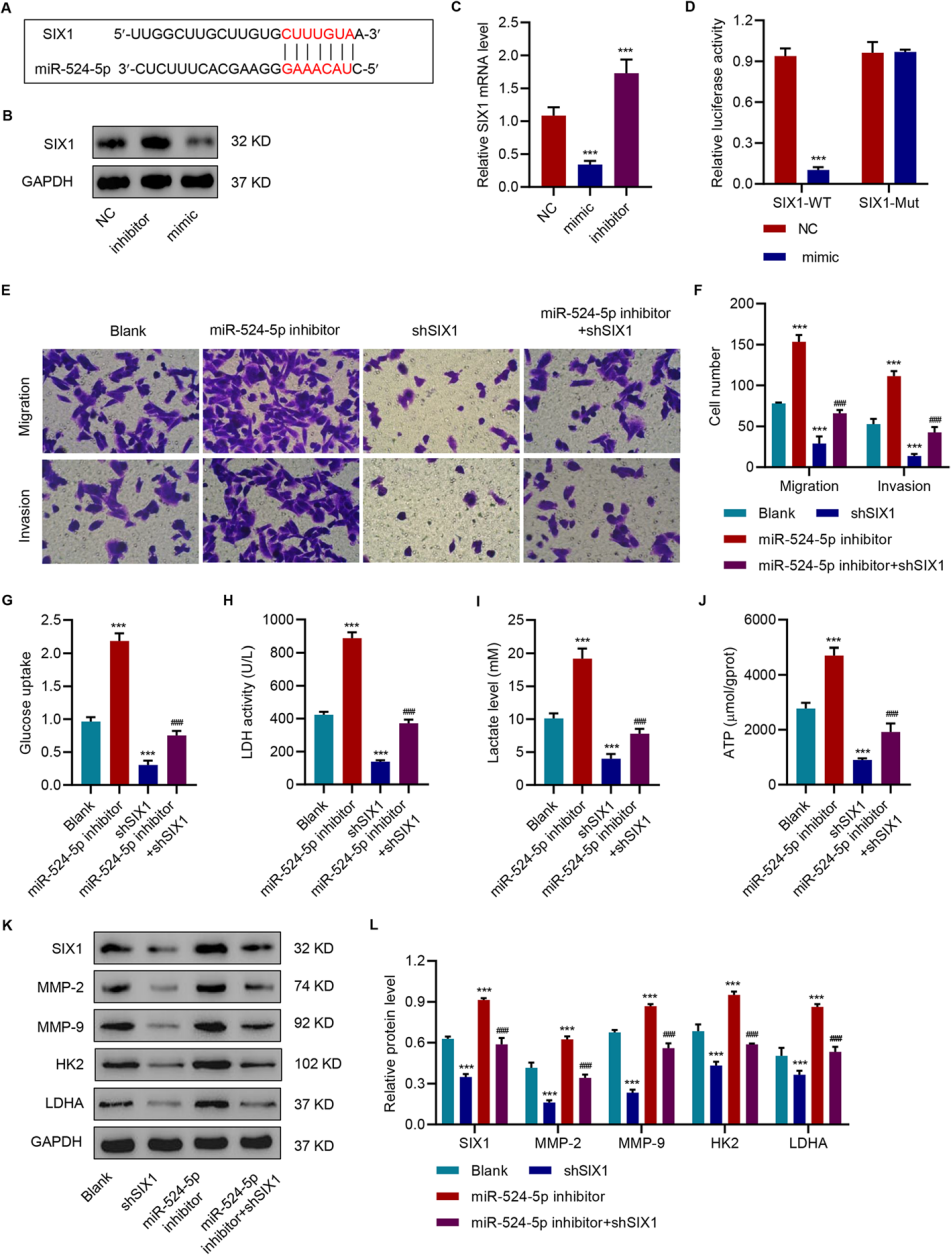
SIX1 knockdown attenuated miR-524-5p inhibitor-mediated promotion of migration, invasion, and glycolysis. **A** The predictive binding of miR-524 and SIX1. **B**, **C** Levels of SIX1 in HepG2 cells with overexpression or silencing of miR-524. **D** miR-524 regulated luciferase activity of SIX1 3′-UTR. Effects of miR-524 inhibitor and SIX1 shRNA on (**E**, **F**) migration/invasion, (**G**) glucose uptake, (**H**) LDH activity, (**I**) lactate, and (**J**) ATP content, and (**K**, **L**) SIX1, MMP-2, MMP-9, HK2, and LDHA. The data are expressed as the mean ± SD (n = 3). ***P < 0.001 vs blank or NC. ^###^*P* < 0.001 vs miR-524 inhibitor

**Fig. 6:**
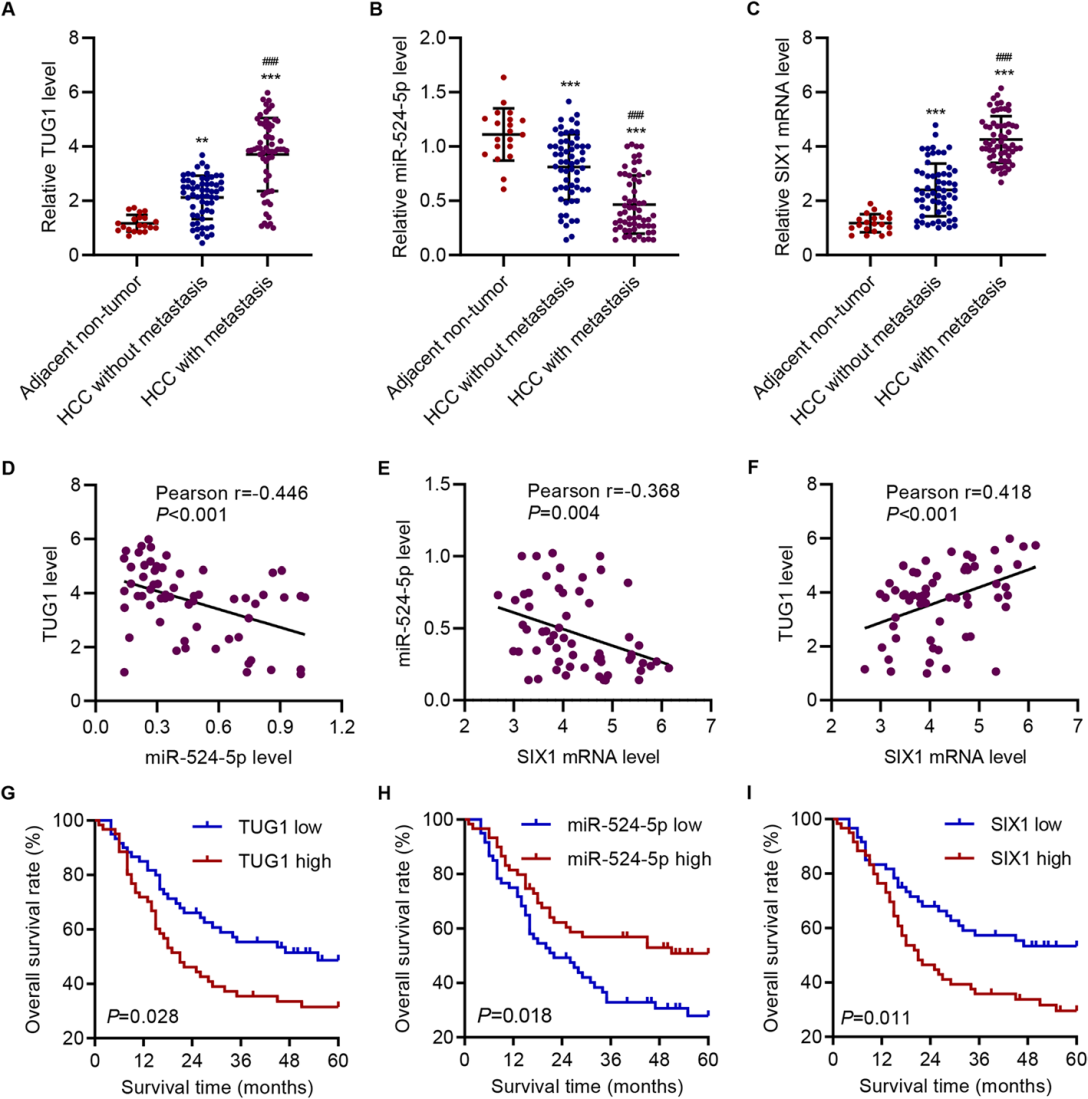
TUG1 negatively correlated with miR-524-5p and positively correlated with SIX1. qPCR assay of (**A**) TUG1, (**B**) miR-524-5p, and (**C**) SIX1 mRNA levels in adjacent non-tumor (n = 20), non-metastatic (n = 60), and metastatic tumor (n = 60) tissues. **D**–**F** Pearson correlation analysis among TUG1, miR-524-5p, and SIX1 mRNA levels in tumor tissues from 60 HCC patients with metastasis. **G**–**I** Survival probability of patients with HCC from hospital cohort, in which patients were split by the median value of TUG1, miR-524-5p, and SIX1 mRNA. The data are expressed as the mean ± SD (n = 20 or 60). **P < 0.01, ***P < 0.001 vs adjacent non-tumor. ^###^*P* < 0.001 vs non-metastatic HCC tissues

To elucidate the mechanism of miR-524-5p, we performed further bioinformatics analysis; the results indicated that miR-524-5p may target SIX1 (Fig. 5A). Overexpression of miR-524-5p reduced SIX1 while inhibition of miR-524-5p enhanced SIX1 expression at both protein and mRNA levels (Fig. 5B, C). We also found that overexpression of miR-524 significantly inhibited the luciferase activity of WT SIX1 (Fig. 5D). Furthermore, silencing of SIX1 abolished the promotive effect of miR-524-5p inhibition on migration, invasion (Figs. 5E, 6F), glucose uptake (Fig. 5G), LDH activity (Fig. 5H), lactate levels (Fig. 5I), ATP levels (Fig. 5J), and MMP-2, MMP-9, HK2, and LDHA in HepG2 (Figs. 5K–L); these findings suggested that miR-524-5p exerted its effects on cell migration, invasion, and glycolysis via suppression of SIX1.

### TUG1 negatively correlated with miR-524-5p but positively correlated with SIX1

To investigate the relationship among TUG1, miR-524, and SIX1, we investigated the levels of TUG1, miR-524, and SIX1 in adjacent non-tumor, non-metastatic HCC, and metastatic HCC tissues. The results showed an increasing trend of TUG1 levels (Fig. 6A), a decreasing trend of miR-524-5p levels (Fig. 6B), and an increasing trend of SIX1 levels (Fig. 6C) in adjacent non-tumor, non-metastatic HCC, and metastatic HCC tissues. Pearson correlation analysis indicated that miR-524-5p decreased with the increase of TUG1 (Fig. 6D) and SIX1 (Fig. 6E). In addition, TUG1 increased with the increase of SIX1 (Fig. 6F). To further explore the clinical relevance of TUG1, tumor samples were characterized as TUG1 low or TUG1 high groups according to the median value for further analyses. Remarkably, TUG1 expression in patients was associated with tumor size (P = 0.032), lung metastasis (P = 0.004), HBV infection (P = 0.024), AJCC stage (P = 0.008), miR-524-5p (P = 0.001), and SIX1 mRNA (P = 0.011) (Additional file 1: Table S1), whereas the expression of TUG1 was not associated with patient age or gender. As shown in Fig. 6G–I, survival rates of patients with high vs. low TUG1, miR-524-5p, and SIX1 mRNA expression showed statistically significant differences, with higher TUG1 and SIX1 expression resulting in lower survival rates (Fig. 6G and I), while higher miR-524-5p expression resulted in higher survival rates (Fig. 6H).

## Discussion

In the present study, CAFs-derived exosomal lncRNA TUG1 promoted migration, invasion, and glycolysis in HepG2, and these effects were attenuated by miR-524; these findings indicate that TUG1/miR-524 may play a role in the progression of HepG2. Our results also showed that miR-524 targeted SIX1; in addition, inhibition of SIX1 abolished the promotive effect of miR-524-5p inhibitor on migration, invasion, and glycolysis. Further mechanistic analysis demonstrated that the TUG1/miR-524-5p/SIX1 axis may regulate HepG2 cell metastasis in vivo.

Accumulating evidence has shown that the cellular interaction between cancer cells and surrounding stroma cells in the tumor microenvironment plays important roles in regulating cancer progression and therapy response [32]. CAFs are key determinants that contribute to growth, invasion, metastasis, and therapy resistance of human cancer by exosome mediated cellular communication [33]. Here, we observed that CAFs secreted exosomes promoting cell migration, invasion, and glycolysis in HCC.

lncRNAs regulate gene expression via various mechanisms [34]. lncRNA CRNDE directly binds to EZH2, SUZ12, and SUV39H1, and mediated their inhibition of tumor suppressor genes, including CELF2 and LATS2 [35]. The promotion of HCC cell metastasis by AY927503 is related to the activation of ITGAV transcription by recruiting chromatin modification mechanisms to the ITGAV promoter and reducing H1FX binding [36]. TUG1 could also upregulate PGC-1α expression through interacting with the upstream region of the PGC-1α gene to promote the binding of PGC1-α to its promoter, which inhibits mitochondrial destruction and facilitates energy metabolism [37]. In the current study, TUG1 expression was increased in HCC patients and higher TUG1 levels were associated with a poor survival outcome. Similarly, previous studies have shown remarkable elevation of TUG1 expression in kidney cancer tissues and its association with shorter overall survival of patients with kidney cancer [38, 39]. However, TUG1 was shown to be downregulated in lung cancer and lower TUG1 level was associated with poorer survival [40]. These results suggested that the function of TUG1 is tissue-specific. Moreover, silencing of TUG1 significantly ameliorated the effects of CAFs-exo in HepG2 cells, indicating that TUG1 promoted the progression of HCC. This is consistent with previous studies, in which upregulation of TUG1 was shown to contribute to tumorigenesis, proliferation, and metastasis of HCC [41, 42].

miR-524-5p has been implicated in different tumor processes [13, 14]. In addition, studies have demonstrated the involvement of miR-524-5p in several other diseases, including hepatic cirrhosis, fibrosis, and amyotrophic lateral sclerosis [43, 44]. A recent study demonstrated elevated expression of miR-524 in colon cancer, which inhibited angiogenesis through WNK1 [14]. In a previous study, overexpression of miR-524 inhibited tumor cell migration [13]. These observations are consistent with our findings, in which upregulation of miR-524-5p was shown to inhibit migration, invasion and glycolysis of HepG2 cells, elucidating a new role of miR-524-5p in HCC. In our study, we found that TUG1 enhanced migration, invasion and glycolysis of HepG2 by acting as a ceRNA and sponging miR-524-5p. This finding is consistent with a previous study indicating that TUG1 could sponge miR-524-5p [23]. To the best of our knowledge, this is the first study showing that TUG1 can interact with miR-524-5p to regulate HepG2 cell growth.

We also confirmed that miR-524-5p targets SIX1. As an important transcription factor, SIX1 plays a very important role in tumorigenesis [45]. Increasing evidence suggests that SIX1 is a tumor promoter [42, 46]. Our data also showed that silencing of SIX1 inhibited migration, invasion and glycolysis of HepG2 cells. Since SIX1 regulates expression of glycolytic genes such as HK2 and LDHA that modulate cell proliferation and/or apoptosis [28], the function of SIX1 in glycolysis at least partly explains these defects induced by SIX1 knockdown. However, we cannot exclude the possibility that other genes regulated by SIX1 may also be responsible for these defects. TUG1 may modulate migration, invasion, and glycolysis in HepG2 cells via regulating the cellular level of miRNA, thereby forming a TUG1/miR-524-5p/SIX1 regulatory axis. However, further studies are required to investigate the involvement of other miRNAs or other miR-524-5p targets.

## Conclusions

To conclude, in this study, CAFs-derived exosomal lncRNA TUG1 promoted migration, invasion, and glycolysis in HepG2 cells through the miR-524-5p/SIX1 axis. The findings indicate an essential role of the TUG1/miR-524-5p/SIX1 axis in HCC.

## Supplementary Information

**Additional file 1. Table S1.** Clinicopathological characteristics and follow-up data of 120 patients with HCC. **Table S2.** Primes sequences used in this study. **Figure S1.** Characterization of CAFs and NFs. Microscopic observation of primary CAFs and NFs. Scale bar: 100 m.**Figure S2.** Identification and internalization of exosomes. (**A**) TEM images of CAFs/NFs-derived exosomes (CAFs-exo/NFs-exo) (scale bar: 200 nm). (**B**) Protein levels of exosomal markers CD63, CD9, and TSG101. (**C**) Confocal microscopic images showing internalization of exosomes by HepG2 cells (scale bar: 50 m).**Figure S3.** Effects of CAFs-derived exosomal TUG1 on HepG2 cell metastasis in vivo. Effects of exosomes derived from the CAFs with or without TUG1 shRNA adenovirus infection on (**A**, **B**) metastasis (scale bar: 200 m) (n = 6) and (**C**) survival duration of mice (n = 15). The data are expressed as the mean + SD (n = 6). ***P < 0.001 vs blank. ###P < 0.001 vs CAFs-exo.**Figure S4**. The effects of CAFs-derived exosomes on HepG2 cells are inhibited by TUG1 knockdown. (**A**, **B**) Migration and invasion, (**C**) glucose uptake, (**D**) LDH activity, (**E**) lactate, and (**F**) ATP content, (**G**) TUG1 expression, and (**H**, **I**) MMP-2, MMP-9, HK2, and LDHA expressions were measured in HepG2 treated with CAFs-exo and transduced with shTUG1 or shNC. The data are expressed as the mean + SD (n = 3). ***P < 0.001 compared with shNC.

## Data Availability

The authors confirm the availability of all data generated or analyzed in this manuscript.

## Abbreviations

TUG1: Taurine upregulated gene 1
HCC: Hepatocellular carcinoma
SIX1: Sine oculis homeobox homolog 1
LDH: Lactate dehydrogenase
ATP: Adenosine triphosphate
CAFs: Cancer-associated fibroblasts
NFs: Adjacent non-tumor fibroblasts
RIP: RNA immunoprecipitation
H&E: Hematoxylin and eosin
TEM: Transmission electron microscopy
SD: Standard deviation
