# Supplementary material for: Exosomal lncRNA TUG1 from cancer-associated fibroblasts promotes liver cancer cell migration, invasion, and glycolysis by regulating the miR-524-5p/SIX1 axis
Source: Cell Mol Biol Lett. 2022 Feb 22;27:17. doi: 10.1186/s11658-022-00309-9 (PMC8903597; doi:10.1186/s11658-022-00309-9)
Supplement: Supplementary file 1 — Additional file 1. Table S1. Clinicopathological characteristics and follow-up data of 120 patients with HCC. Table S2. Primes sequences used in this study. Figure S1. Characterization of CAFs and NFs. Microscopic observation of primary CAFs and NFs. Scale bar: 100 m.Figure S2. Identification and internalization of exosomes. (A) TEM images of CAFs/NFs-derived exosomes (CAFs-exo/NFs-exo) (scale bar: 200 nm). (B) Protein levels of exosomal markers CD63, CD9, and TSG101. (C) Confocal microscopic images showing internalization of exosomes by HepG2 cells (scale bar: 50 m).Figure S3. Effects of CAFs-derived exosomal TUG1 on HepG2 cell metastasis in vivo. Effects of exosomes derived from the CAFs with or without TUG1 shRNA adenovirus infection on (A, B) metastasis (scale bar: 200 m) (n = 6) and (C) survival duration of mice (n = 15). The data are expressed as the mean + SD (n = 6). ***P < 0.001 vs blank. ###P < 0.001 vs CAFs-exo.Figure S4. The effects of CAFs-derived exosomes on HepG2 cells are inhibited by TUG1 knockdown. (A, B) Migration and invasion, (C) glucose uptake, (D) LDH activity, (E) lactate, and (F) ATP content, (G) TUG1 expression, and (H, I) MMP-2, MMP-9, HK2, and LDHA expressions were measured in HepG2 treated with CAFs-exo and transduced with shTUG1 or shNC. The data are expressed as the mean + SD (n = 3). ***P < 0.001 compared with shNC. [file 11658_2022_309_MOESM1_ESM.docx]

**Table S1.** Clinicopathological characteristics and follow-up data of 120 patients with HCC

| Characteristics | TUG1 expression | | *P* value |
| --- | --- | --- | --- |
|  | Low | High |  |
| **Gender** |  |  | 0.258 |
| Male (n=75) | 34 | 41 |  |
| Female (n=45) | 26 | 19 |  |
| **Age (years)** |  |  | 0.273 |
| ≤52 (n=57) | 25 | 32 |  |
| >52 (n=63) | 35 | 28 |  |
| **Tumor size (cm)** |  |  | 0.032 |
| ≤5 (n=39) | 25 | 14 |  |
| >5 (n=81) | 35 | 46 |  |
| **Lung metastasis**  Yes (n=60)  No (n=60) | 22  38 | 38  22 | 0.004 |
| **HBV*** **infection**  Yes (n=74)  No (n=46) | 31  29 | 43  17 | 0.024 |
| **AJCC stage**  I (n=25)  II (n=46)  III (n=34)  IV (n=15) | 20  20  15  5 | 5  26  19  10 | 0.008 |
| **miR-524-5p**  Low  High | 21  39 | 39  21 | 0.001 |
| **SIX1 mRNA** |  |  | 0.011 |
| Low | 37 | 23 |  |
| High | 23 | 37 |  |

*HBV, hepatitis B virus.

Differences between groups were determined by the Chi-square test.

**Table S2.** Primes sequences used in this study

| Gene | Sequences (5′-3′) |
| --- | --- |
| TUG1-forward  TUG1-reverse  GAS5-forward  GAS5-reverse  H19-forward  H19-reverse  MALAT1-forward  MALAT1-reverse  MEG3-forward  MEG3-reverse  NEAT1-forward  NEAT1-reverse  XIST-forward  XIST-reverse  SIX1-forward  SIX1-reverse  GAPDH-forward  GAPDH-reverse  miR-524-5p-forward  miR-524-5p-reverse  U6-forward  U6-reverse | CAGCAAATCCATCTGAAC  ACTGGCTTCATTCTCTAC  CACAGGCATTAGACAGAAAGC  TCCTTACCCAAGCAAGTCATC  GCGGGTCTGTTTCTTTACTTCC  CTTTGATGTTGGGCTGATGAGG  TTTCTTCCTGCTCCGGTTC  TTTCAGCTTCCAGGCTCTC  CTGGGTCGGCTGAAGAACTG  AGGGCGGGTCTCTACTCAAG  CCTCCCTTTAACTTATCCATTC  TCCACCATTACCAACAATAC  CTACCGCTTTGGCAGAGAATG  GCCTCCCGATACAACAATCAC  AAGGTGAGTGGTGTATTG  TGCTGTGAAGAGATAGTG  AATCCCATCACCATCTTC  AGGCTGTTGTCATACTTC  GCGCTACAAAGGGAAGCAC  AGTGCAGGGTCCGAGGTATT  CTCGCTTCGGCAGCACA  AACGCTTCACGAATTTGCGT |


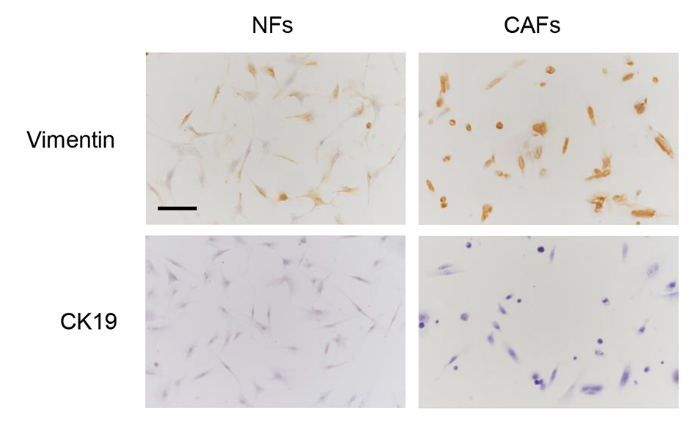


**Figure S1.** **Characterization of CAFs and NFs.** Microscopic observation of primary CAFs and NFs. Scale bar: 100 μm.


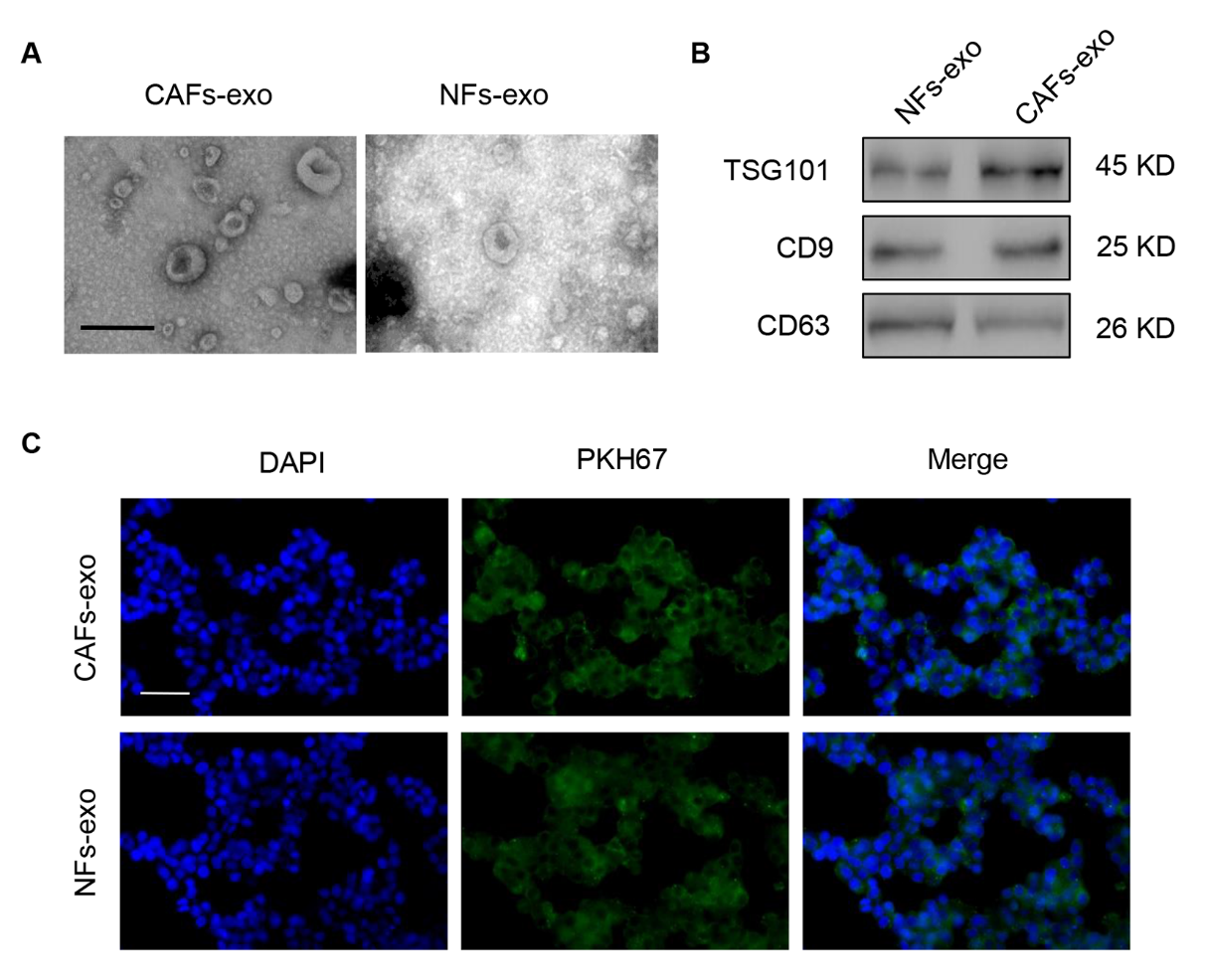


**Figure S2.** **Identification and internalization of exosomes.** (A) TEM images of CAFs/NFs-derived exosomes (CAFs-exo/NFs-exo) (scale bar: 200 nm). (B) Protein levels of exosomal markers CD63, CD9, and TSG101. (C) Confocal microscopic images showing internalization of exosomes by HepG2 cells (scale bar: 50 μm).


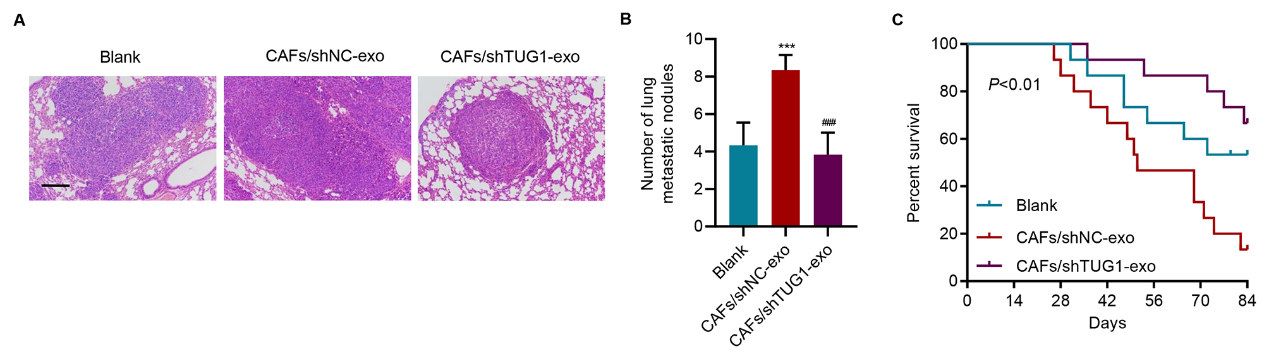


**Figure S3. Effects of CAFs-derived exosomal TUG1 on HepG2 cell metastasis *in vivo*.** Effects of exosomes derived from the CAFs with or without TUG1 shRNA adenovirus infection on (A, B) metastasis (scale bar: 200 μm) (n = 6) and (C) survival duration of mice (n = 15). The data are expressed as the mean + SD (n = 6). ****P* < 0.001 vs blank. ^###^*P* < 0.001 vs CAFs-exo.


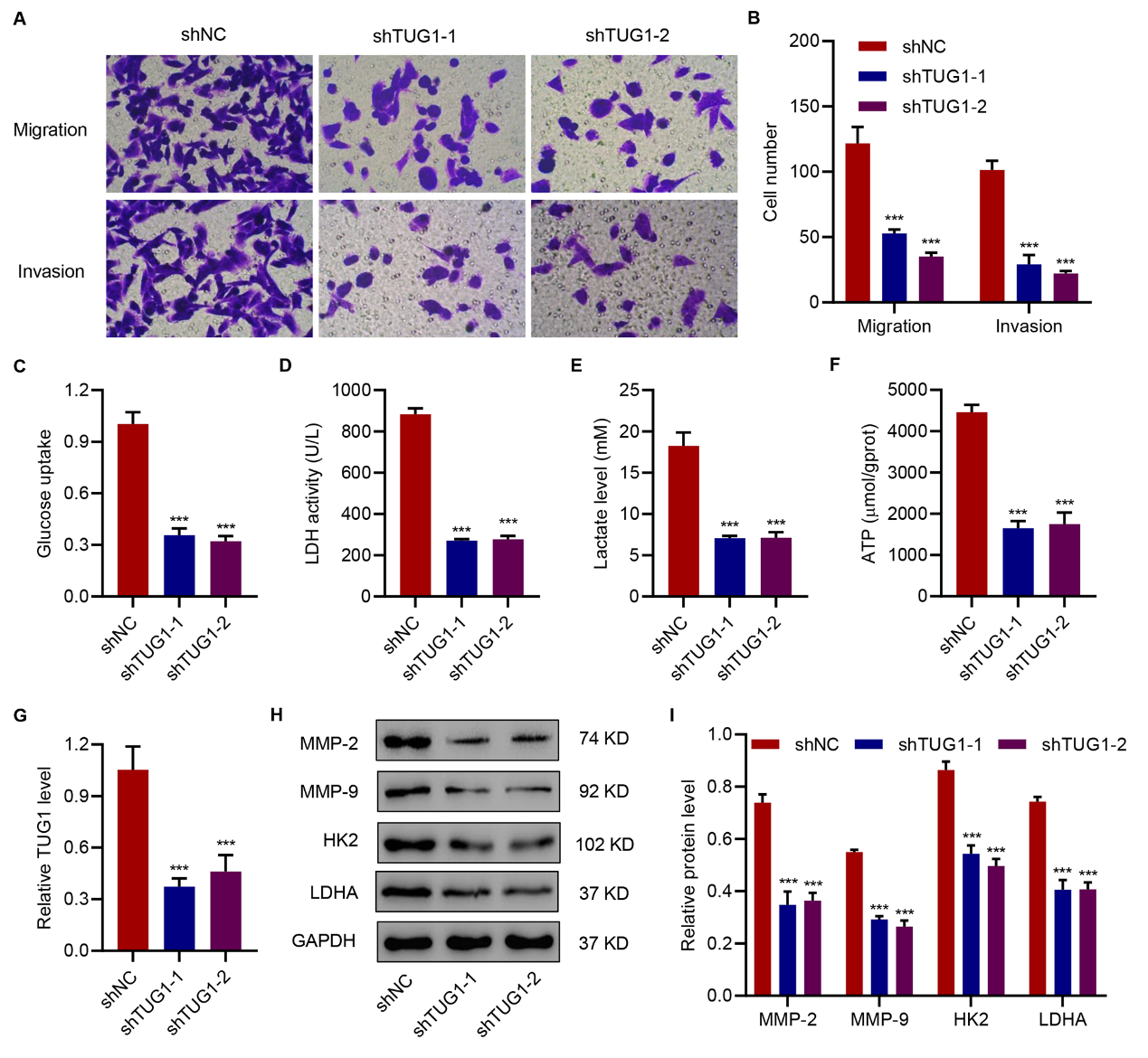


**Figure S4. The effects of CAFs-derived exosomes on HepG2 cells are inhibited by TUG1 knockdown.** (A, B) Migration and invasion, (C) glucose uptake, (D) LDH activity, (E) lactate, and (F) ATP content, (G) TUG1 expression, and (H, I) MMP-2, MMP-9, HK2, and LDHA expressions were measured in HepG2 treated with CAFs-exo and transduced with shTUG1 or shNC. The data are expressed as the mean + SD (n = 3). ****P* < 0.001 compared with shNC.
